# Genetic variability and population structure of endangered *Panax ginseng *in the Russian Primorye

**DOI:** 10.1186/1749-8546-5-21

**Published:** 2010-06-11

**Authors:** Yuri N Zhuravlev, Galina D Reunova, Irina L Kats, Tamara I Muzarok, Alexander A Bondar

**Affiliations:** 1 Department of Biotechnology, Institute of Biology and Soil Science of the Russian Academy of Sciences, Vladivostok, 690022, Russia; 2 Institute of Chemical Biology and Fundamental Medicine, Novosibirsk, 630090, Russia

## Abstract

**Background:**

The natural habitat of wild *P. ginseng *is currently found only in the Russian Primorye and the populations are extremely exhausted and require restoration. Analysis of the genetic diversity and population structure of an endangered species is a prerequisite for conservation. The present study aims to investigate the patterns and levels of genetic polymorphism and population structures of wild *P. ginseng *with the AFLP method to (1) estimate the level of genetic diversity in the *P. ginseng *populations in the Russian Primorsky Krai, (2) calculate the distribution of variability within a population and among populations and (3) examine the genetic relationship between the populations.

**Methods:**

Genetic variability and population structure of ten *P. ginseng *populations were investigated with Amplified Fragment Length Polymorphism (AFLP) markers. The genetic relationships among *P. ginseng *plants and populations were delineated.

**Results:**

The mean genetic variability within populations was high. The mean level of polymorphisms was 55.68% at the population level and 99.65% at the species level. The Shannon's index ranged between 0.1602 and 0.3222 with an average of 0.2626 at the population level and 0.3967 at the species level. The analysis of molecular variances (AMOVA) showed a significant population structure in *P. ginseng*. The partition of genetic diversity with AMOVA suggested that the majority of the genetic variation (64.5%) was within populations of *P. ginseng*. The inter-population variability was approximately 36% of the total variability. The genetic relationships among *P. ginseng *plants and populations were reconstructed by Minimum Spanning tree (MS-tree) on the basis of Euclidean distances with ARLEQUIN and NTSYS, respectively. The MS-trees suggest that the southern *Uss*, *Part *and *Nad *populations may have promoted *P. ginseng *distribution throughout the Russian Primorye.

**Conclusion:**

The *P. ginseng *populations in the Russian Primorye are significant in genetic diversity. The high variability demonstrates that the current genetic resources of *P. ginseng *populations have not been exposed to depletion.

## Background

*Panax ginseng *C.A. Meyer (*Renshen*, Asian ginseng) is a representative species of the *Panax *L. genus which is a relic of the Araliacea family [1]. Their natural stocks are over-exploited because they have the highest biological activities [2]. At the beginning of the twentieth century, wild *P. ginseng *spread over a vast territory including the Russian Primorsky Krai, Korea and China. Currently, wild *P. ginseng *can only be found in Russia; however, its populations are extremely exhausted and restoration is needed [1]. *P. ginseng *is listed in the Red Book of Primorsky Krai as an endangered species [3].

Analysis of the genetic diversity and population structure of an endangered species is a prerequisite for conservation [4]. Genetic variability is critical for a species to adapt to environmental changes and survive in the long term. A species with little genetic variability may suffer from reduced fitness in its current environment and may not have the evolutionary potential necessary for a changing environment [5]. Knowledge of genetic diversity within a population and among populations is important for conservation management, especially in identifying genetically unique structural units within a species and determining the populations that need protection.

A high level of polymorphism of a marker is a basic condition that must be assessed population genetics studies [6]. A study using allozyme analysis found a low level of polymorphism (7%) in wild ginseng [7]. Multi-locus DNA markers, e.g., Random Amplified Polymorphic DNA (RAPD), Inter Simple Sequence Repeat (ISSR) and Amplified Fragment Length Polymorphism (AFLP) would potentially produce higher values of polymorphism than allozyme analysis because non-coding DNA sequences, which mutate at a higher speed than coding sequences, would also be characterized [8]. RAPD polymorphisms in wild ginseng populations are low [7,9]. Results with RAPD markers corresponded with the lack of genetic variation demonstrated by isozyme gene loci in red pine [10]. In contrast, polymorphism in RAPD loci (about 46%) is high in cultivated *P. ginseng *[11]. Allozymes and RAPD markers are highly variable in populations of *Panax quinquefolius *(*Xiyangshen*, American ginseng) [12-16]. There are 62.5% polymorphic loci in populations of *P. quinquefolius *in the United States [16]. *P. quinquefolius *population from Ontario, Canada, has a polymorphism level of about 46% estimated with RAPD analysis [14].

As a reproducible and robust technique, AFLP [17] generates a large number of bands per assay and is best suited for analyzing genetic diversity. The fluorescence-based automated AFLP method demonstrated the highest resolving power as a multi-loci technique [18-20]. An automated DNA fingerprinting system utilizing fluorescently labeled primers and the laser detection technology associated with the automatic sequencer allowed the resolution of fragments that were undistinguishable by other methods. In a previous study, four fluorescently labeled AFLP primer pairs and 20 RAPD primers generated 645 and 170 polymorphic markers respectively [18]. In a study to characterize *Miscanthus*, three fluorescently labeled AFLP primer pairs generated 998 polymorphic markers, as opposed to only 26 polymorphic markers produced by two ISSR [20].

The present study aims to investigate the patterns and levels of genetic polymorphism and population structures of wild *P. ginseng *with the AFLP method to (1) estimate the level of genetic diversity in the *P. ginseng *populations in the Russian Primorsky Krai, (2) calculate the distribution of variability within a population and among populations and (3) examine the genetic relationship between the populations.

## Methods

### Sampled populations

**Figure 1:**
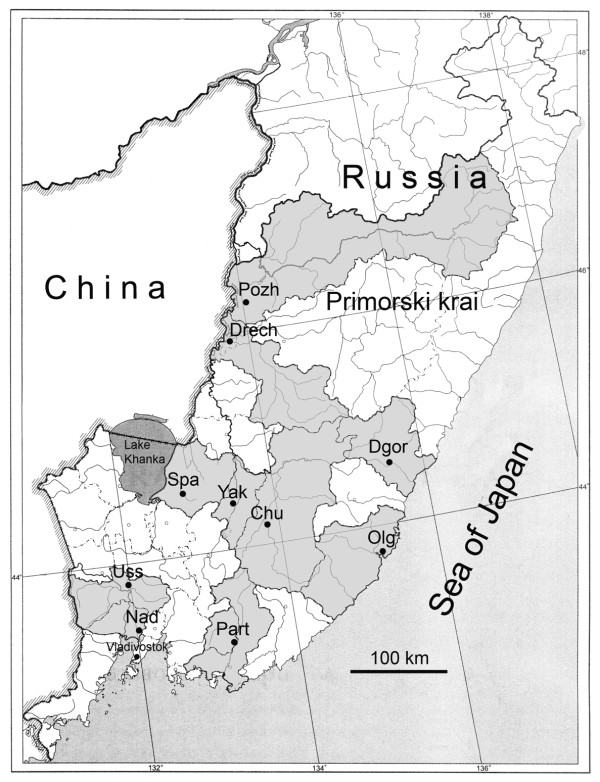
**The administrative areas in the territory of the Russain Primorskiy Krai where *Panax ginseng *plants were collected**.

One hundred and sixty-seven (167) *P. ginseng *individuals were collected from the ten administrative areas of Primorsky Krai (Figure 1) and transferred to a collection nursery. The study populations were coded with the names of the areas. Twenty (20) *P. ginseng *individuals were collected from the Chuguevsk area (*Chu*), 19 from the Spassk area (*Spa*), 16 from the Ussuriisk area (*Uss*), 13 from the Dalnerechensk area (*Drech*), 16 from the Dalnegorsk area (*Dgor*), 15 from the Olginsk area (*Olg*), 15 from the Pozharsk area (*Pozh*), 24 from the Nadezhdinsk area (*Nad*), 19 from the Partizansk area (*Part*) and 10 from the Yakovlevsk area (*Yak*).

### DNA extraction

Total genomic DNA was extracted from fresh leaf tissue according to Echt *et al*. [21]. The extracted DNA was purified according to the Murray and Thompson method [22].

### AFLP procedure

AFLP genotyping was performed according to Vos *et al*. [17] using *Eco*RI and *Mse*I restriction enzymes. Pre-amplification reactions utilized AFLP primers with two selective nucleotides. *Eco*RI and *Mse*I selective amplification primers contained three and four selective nucleotides, respectively (Table 1). AFLP adapters and primers were purchased from Syntol (Russia). All the *Eco*RI-NNN selective primers were labeled with fluorescent 6-carboxy fluorescein (6-FAM) at the 5' end. The AFLP fragments were analyzed on an ABI Prism 3100 automated capillarity system with GeneScan Analysis Software (Applied Biosystems, USA). All unambiguous peaks including monomorphic peaks between 50-500 base pairs (bp) were analyzed and the scoring results were exported as a presence/absence matrix.

**Table 1:** AFLP selective primers used in the study of the population genetics of *Panax ginseng*

*Eco *RI primer	*Mse *I primer	Number of loci
*E-ACA*	*M-CCGG*	149
*E-ACA*	*M-CCTG*	133
Total		282

### Data analysis

Parameters of genetic variability and genetic mutual relations of populations were calculated with the POPGEN32 (POPGENE v. 1.31, Centre for International Forestry Research, University of Alberta and Tim Boyle, Canada) [23] and ARLEQUIN (Arlequin v.3.11, Excoffier L. Zoological Institute, University of Berne, Switzerland). As AFLPs were dominant markers, Shannon's information measure (*I*_S_) [24] was used to quantify the degree of the within-population diversity. Analysis of molecular variance (AMOVA) [25] was conducted to calculate the variance components and significance levels of variation within a population and among populations. AMOVA derived genetic differentiation values (*F*_ST_) between pairs of populations (analogous to traditional *F *statistics) were calculated. Gene flow between pairs of populations (*N*_m _= (1-*F*_ST_)/4*F*_ST_) was calculated from *F*_ST _values [26]. We reconstructed the Minimum Spanning tree (MS-tree) between representatives of *P. ginseng *and populations from a matrix of squared Euclidean distances using ARLEQUIN (Arlequin v.3.11, Excoffier L. Zoological Institute, University of Berne, Switzerland) and NTSYS (NTSYS-pc v.1.70, Applied Biostatistics, Inc, USA) respectively.

## Results

Nine (9) AFLP primer pairs were tested, namely *Eco*(ACG)/*Mse*(CCTC), *Eco*(ACG)/*Mse*(CCTT), *Eco*(ACA)/*Mse*(CCTG), *Eco*(ACA)/*Mse*(CCGG), *Eco*(ACA)/*Mse*(CCAC), *Eco*(ACT)/*Mse*(CCGA), *Eco*(ACT)/Mse(CCTA), *Eco*(ACC)/*Mse*(CCAG), and *Eco*(ACC)/*Mse*(CCGC). Using two of the primer pairs *Eco *(ACA)/*Mse*(CCTG) and *Eco*(ACA)/*Mse*(CCGG) (Table 1), we detected polymorphic bands among the various samples of *P. ginseng *in this study. Among the scored 282 fragments, 281 were polymorphic across all ten populations (Table 2). Genetic variability was high within populations (Table 2). The highest genetic diversity values (approximately 70%) were obtained in the *Chu, Nad, Olg *and *Pozh *populations, whereas the lowest values (approximately 40%) were found in the *Uss *and *Dgor *populations. The mean level of polymorphisms was 55.68% at the population level and 99.65% at the species level. The Shannon's index ranged between 0.1602 and 0.3222 with an average of 0.2626 at the population level and 0.3967 at the species level. The intra-population genetic polymorphisms ranged from 38.65% (*Uss*) to 69.15% (*Chu*) with an average of 55.68% (Table 2).

**Table 2:** Sample size and genetic variability parameters of *Panax ginseng *populations calculated from AFLP data for 282 fragments

Population number	Population code	Number of plants (order numbers of plants)	Shannon's index (*I*_S_)	Polymorphic loci	
				Number	% (*P*)
1	*Spa*	19 (17-35)	0.2972	163	57.80
2	*Yak*	10 (158-167)	0.2487	145	51.42
3	*Drech*	13 (36-48)	0.2614	138	48.94
4	*Pozh*	15 (100-114)	0.3222	186	65.96
5	*Uss*	16 (1-16)	0.1602	109	38.65
6	*Nad*	24 (115-138)	0.2840	190	67.38
7	*Chu*	20 (49-68)	0.3169	195	69.15
8	*Dgor*	16 (69-84)	0.1821	114	40.43
*9*	*Olg*	15 (85-99)	0.3195	188	66.67
10	*Part*	19 (139-157)	0.2335	142	50.35
Population average		17	0.2626	157	55.68
Species-level value		167	0.3967	281	99.65

All pair wise *F*_ST _between populations, obtained with AMOVA, were significant (*P *= 0.0000) and varied from 0.09180 (*Pozh-Nad*) to 0.60506 (*Drech-Uss*) (Table 3). The non-hierarchical AMOVA analyses revealed that 35.54% of the total variation was attributed to the variability among the populations, whereas 64.46% was accumulated within the populations (Table 4). The average number of migrants (*N*_m_) between populations based on AMOVA (*F*_ST _= 0.355) was 0.45.

**Table 3:** Matrix of pairwise differences (*F*_ST_) among *Panax ginseng *populations calculated with AMOVA

	1	2	3	4	5	6	7	8	9	10
1	0.00000									
2	0.41235	0.00000								
3	0.27212	0.53153	0.00000							
4	0.30808	0.26936	0.47046	0.00000						
5	0.35629	0.52259	0.60506	0.36954	0.00000					
6	0.30464	0.25556	0.49335	0.09180	0.36057	0.00000				
7	0.18200	0.42200	0.21348	0.35356	0.40031	0.35103	0.00000			
8	0.21894	0.48054	0.54275	0.32029	0.27451	0.31409	0.33000	0.00000		
9	0.38764	0.25381	0.42708	0.24434	0.49424	0.30650	0.35041	0.46318	0.00000	
10	0.34993	0.16600	0.52375	0.27691	0.42249	0.15721	0.38540	0.39335	0.36194	0.00000

P value = 0.00000

**Table 4:** AMOVA analysis of genetic variances within and among populations of *Panax ginseng *(Level of significance is based on 1000 iterations)

Source of variation	Degree of freedom	Sum of squares	Variance components	Percentage of variation
Among populations	9	2413.140	14.55557	35.54
Within populations	157	4145.651	26.40542	64.46
Total	166	6558.790	40.96099	

Fixation index *F*_ST _= 0.35535

P value = 0.0000

**Figure 2:**
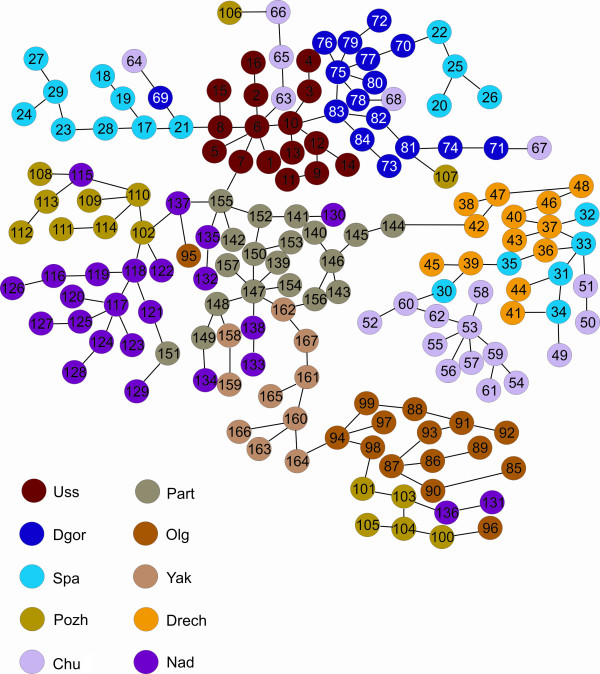
**MS-tree representing phylogenetic relationships among representative *Panax ginseng *populations**. Length of lines is proportional to the Euclidean distances among plants. Length of scale line is equal to 50 units of Euclidean distances

The MS-tree showed the genetic relationships among *P. ginseng *plants (Figure 2). Calculated in AMOVA on the basis of Euclidean distances, the length of the lines connecting the representatives inside the populations and between the populations reflects the intra- and inter-population genetic distances respectively (Table 5).

**Table 5:** The length of lines on MS-tree characterizing the Euclidean genetic distances among plants in populations and among populations of *Panax ginseng*

Among plants in population			Among populations	
Population	Range of length	Average length	Population pair	Length
*Uss*	8-41	17.33	*Uss - Spa*	24
*Spa*	9-30	22.57	*Uss - Dgor*	22
*Dgor*	15-38	23.0	*Uss - Part*	33
*Pozh*	14-43	28.0	*Part - Drech*	50
*Nad*	21-36	29.07	*Drech - Chu*	25
*Part*	12-30	22.06	*Part - Yak*	19
*Yak*	22-57	35.13	*Yak - Olg*	35
*Chu*	15-44	25.0	*Pozh -Nad*	24
*Drech*	12-51	24.88	*Pozh - Part*	27
*Olg*	14-52	36.5		
Average		26.35		28.78

According to values of genetic distances, all of the studied ginseng plants on the MS-tree formed two groups (Figure 2, Table 5), the first group consisting of the *Drech *and *Chu *populations and the second group the *Part*, *Yak*, *Olg*, *Nad*, *Pozh*, *Uss*, *Dgor *and *Spa *populations. These two groups were divided by a genetic distance of 50 units of Euclidean distance (Figure 2, Table 5). The *Spa*, *Uss*, *Dgor *and *Part*, *Yak*, *Nad*, *Pozh *populations formed two subgroups divided by a genetic distance of 33 Euclidean distance units. The plants of the *Olg *population were distanced from the *Part*, *Yak*, *Nad*, *Pozh *subgroup by 35 Euclidean distance units (Figure 2, Table 5).

The location of a *P. ginseng *on the MS-tree was dependent on the population it belonged to; however, such clustering was not strict and some populations partially overlapped (Figure 2). For example, some plants of the *Pozh *population were grouped with those of the *Olg *population while some plants of the *Spa *population were with the *Dgor *and *Drech *populations. The plants of the *Nad *population were partially mixed with those of the *Part *and *Pozh *populations. Moreover, the plants of the *Chu *population were mixed with those of the *Uss*, *Drech *and *Dgor *populations.

**Figure 3:**
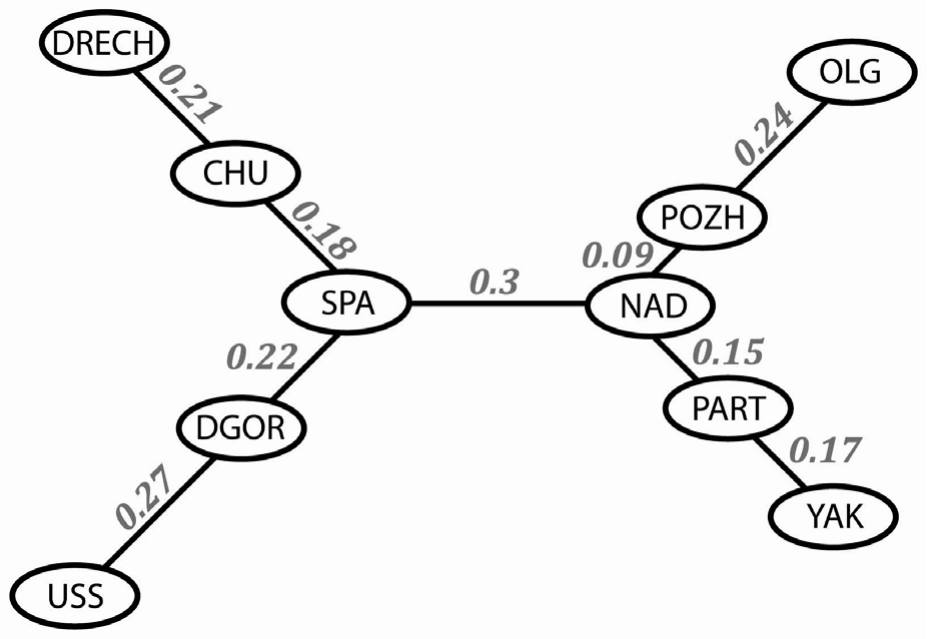
**MS-tree representing phylogenetic relationships among *Panax ginseng *populations**. The numbers on lines show the genetic *F*_ST _distances among populations.

The arrangement of the populations on the MS-tree did not always correspond to their geographical areas. For example, the *Pozh *population was geographically distant from the *Nad *and *Part *populations but was genetically close to them (Figure 2 and 3, Table 5). By contrast, populations that are geographically close, such as *Uss *and *Nad*, were genetically distant and therefore belonged to different subgroups (Figure 2) or groups (Figure 3).

The *Uss *population was characterized by the smallest average value of Euclidean genetic distances between plants (17.33 units), whereas the *Olg *population was characterized by the highest value (36.5 units). The average value of Euclidean genetic distances between the plants of different populations (28.78 units) was higher than that of intra-population genetic distances (26.35 units) (Table 5).

## Discussion

*P. ginseng *populations located in Primorsky Krai have a low level of genetic polymorphisms (approximately 7%) by allozyme and RAPD [7,9,27-29] which means effective conservation strategies would be difficult to implement.

High genetic variability in *P. ginseng *was revealed by the AFLP method. While genetic diversity is theoretically higher in large populations, the *Uss *population was small in size but appeared to have suffered from the loss of a genetic diversity more than other populations. Several populations (*Spa*, *Pozh*, *Nad*, *Chu *and *Olg*) were distinguished by having higher levels of variability. For these populations, the average value of polymorphisms was 65.39%. At the species level, the percentage of polymorphisms was 99.65%. The high level of variability may be due to cross-pollination; however, *P. ginseng*'s capability for cross-pollination is yet to be established [30]. A large number of the insects visiting *P. ginseng *inflorescences are potential pollinators [1]. In *Panax notoginseng*, four pairs of fluorescently labeled AFLP primers produced 312 fragments, of which 240 (76.9%) were polymorphic [31]. In *Panax stipuleanatus*, the same primers revealed 346 loci, of which 334 (96.5%) were polymorphic [31].

Analysis of molecular variance (AMOVA) of the AFLP data showed a significant population pattern of the wild Russian *P. ginseng*. *F*_ST_, estimates of inter-population variability, varied from 0.09180 to 0.60506 (Table 3), indicating that all populations may be different from each other. The partition of genetic diversity with AMOVA suggested that the majority of the genetic variation (64.5%) was within populations of *P. ginseng*. The inter-population variability was approximately 36% of the total variability (Table 4). The value of gene flow (*N*_m_) was 0.45; therefore, wild *P. ginseng *has a relatively high genetic differentiation value among populations and a relatively low level of gene flow. In cultivated *P. ginseng*, inter-population RAPD variability ranged from 1.77% to 42.01% [11] and was 31% in another study [32]. The fluorescence-based automated AFLP method demonstrated that over 40% of the genetic variation of wild *P. stipuleanatus *was among the populations [31]. *P. ginseng*' *F*_ST _values are consistent with estimates of inter-population variability, which were obtained with AMOVA and AFLP markers for plant species with mixed type of propagation (*F*_ST _= 0.35) [33]. According to Nybom [33], *P. ginseng *is a species with mixed type of propagation. The ability of *P. ginseng *to produce seeds via autogamy, out-crossing or agamospermy without pollination was demonstrated earlier [30]. The high level of genetic variation and high proportion of variation within populations in *P. ginseng *suggest that human activities (e.g. overexploitation, habitat destruction, urbanization, pollution) are the major contributor that threatens the survival of the wild *P. ginseng *populations.

Six populations (*Uss*, *Part*, *Olg*, *Yak*, *Dgor *and *Drech*) clustered together and four populations (*Spa*, *Chu*, *Pozh *and *Nad*) were partially mixed with other populations (Figure 2). We believe that the spread of wild *P. ginseng *seeds by humans, animals and birds is the main factor contributing to the population re-mixing.

The MS-tree arrangement of populations did not always correspond to their geographical areas, which may be due to converging common selection forces in geographically disparate populations [34]. Future research with greater numbers of AFLP loci coupled with other high variable markers (SSR) is warranted to confirm the factors that shaped the genetic structures of *P. ginseng *in Russia.

The finding that the average value of inter-population genetic distances is higher that of intra-population genetic distances (Table 5) is consistent with the AMOVA conclusion that reveals the population genetic structures of wild *P. ginseng*.

The *Uss *population was characterized by the least average value of genetic distances between plants (Table 5), which was consistent with the low parameters of variability calculated in POPGENE for this population (Table 2),. On the other hand, the *Olg *population demonstrated the highest genetic distances (Table 5). The *Olg *population is, therefore, the most genetically diverse population according to the MS-tree, suggesting that it should be conserved first.

The central node position on the MS-tree is occupied by a plant (No. 6) that belongs to the *Uss *population and the genetic communications spread to the *Spa *and *Dgo*r populations, and to a cluster of the rest of the *P. ginseng *populations (*Part*, *Nad*, *Yak*, *Olg*, *Chu*, *Drech *and *Pozh*), suggesting the ancestral status of the *Uss *population. The *Part *population, also at the central position on the MS-tree, may have the same ancestral status as the *Uss *population (Figure 2); *Nad *and *Spa *populations may be ancestors as well (Figure 3). The absence of a strong *Spa *population cluster on the MS-tree (Figure 2) may be evidence for its ancestral origin.

The MS-trees suggest that the southern *Uss*, *Part *and *Nad *populations may have promoted *P. ginseng *distribution throughout the Russian Primorye. This result supports the assumption that Sikhote-Alin was re-colonized by *P. ginseng *when thermophilic plants spread from the south to the north during the early Holocene warm period [27].

Future studies may focus on (1) using AMOVA to investigate whether genetically differentiated regions exists for *P. ginseng *and whether *P. ginseng *is adapted for heterogeneous conditions; (2) whether a positive correlation between genetic and geographical distances among *P. ginseng *populations may be established; and (3) using the multi-locus mating system program (MLTR) to estimate the level of inbreeding and cross-pollination in wild *P. ginseng *populations.

## Conclusion

The *P. ginseng *populations in the Russian Primorye contain a significant level of genetic diversity and are essentially differentiated. The gene flow of the populations was less than one (*N*_m _= 0.45) which indicates continued divergence among populations [26]. The current high level of variability demonstrates that the genetic resources of *P. ginseng *populations have not been exposed to depletion.

## Abbreviations

AFLP: Amplified Fragment Length Polymorphism; ISSR: Inter Simple Sequence Repeat; AFLP: Amplified Fragment Length Polymorphism; *Chu*: Chuguevsk area; *Spa*: Spassk area; *Uss*: Ussuriisk area; *Drech*: Dalnerechensk area; *Dgor*: Dalnegorsk area; *Olg*: Olginsk area; *Pozh*: Pozharsk area; *Nad*: Nadezhdinsk area; *Part*: Partizansk area; *Yak*: Yakovlevsk area; bp: base pairs; AMOVA: Analysis of molecular variance; MS-tree: Minimum Spanning tree; 6-FAM: 6-carboxy fluorescein

## Competing interests

The authors declare that they have no competing interests.

## Authors' contributions

YNZ and GDR designed the research. GDR and ILK performed the research and analyzed the data. TIM collected the plants. GDR wrote the manuscript. AAB contributed to the data acquisition. YNZ helped in writing the manuscript and coordinating the study. All authors read and approved the final version of the manuscript.
